# Identification of the point of diminishing returns in high-multiplicity data collection for sulfur SAD phasing

**DOI:** 10.1107/S1600577516014764

**Published:** 2017-01-01

**Authors:** Selina L. S. Storm, Fabio Dall’Antonia, Gleb Bourenkov, Thomas R. Schneider

**Affiliations:** a Hamburg Outstation c/o DESY, European Molecular Biology Laboratory, Notkestrasse 85, 22603 Hamburg, Germany

**Keywords:** anomalous diffraction, radiation damage, SAD-phasing, sulfur

## Abstract

High-quality high-multiplicity X-ray diffraction data were collected on five different crystals of thaumatin using a homogeneous-profile X-ray beam at *E* = 8 keV to investigate the counteracting effects of increased multiplicity and increased radiation damage on the quality of anomalous diffraction data collected on macromolecular crystals. By comparing sulfur substructures obtained from subsets of the data selected as a function of absorbed X-ray dose with sulfur positions in the respective refined reference structures, the doses at which the highest quality of anomalous differences could be obtained were identified for the five crystals. A statistic σ{Δ*F*}_*D*_, calculated as the width σ of the normalized distribution of a set {Δ*F*} of anomalous differences collected at a dose *D*, is suggested as a measure of anomalous data quality as a function of dose. An empirical rule is proposed to identify the dose at which the gains in data quality due to increased multiplicity are outbalanced by the losses due to decreases in signal-to-noise as a consequence of radiation damage. Identifying this point of diminishing returns allows the optimization of the choice of data collection parameters and the selection of data to be used in subsequent crystal structure determination steps.

## Abstract 2

A statistic based on the distribution of sets of signed anomalous differences is evaluated as a possible metric for defining the point at which the inclusion of additional data from a progressively more damaged crystal into a S-SAD phasing process will reduce the probability of successful phasing.

## 1. Introduction   

Single-anomalous dispersion phasing (SAD) exploiting the anomalous signal from sulfur atoms (S-SAD) has become a widely applied method in macromolecular crystallography (Hendrickson, 2014 ▸; Liu & Hendrickson, 2015 ▸). An important advantage of S-SAD phasing is that the S atoms providing the anomalous signal are naturally present in many protein molecules obviating the need to introduce anomalous scatterers such as selenium or metal atoms into the crystal. The expected anomalous signal is generally in the few percent range and therefore can be difficult to measure. However, with recent advances in data collection technologies and computational methods, such small signals are detectable and usable.

It has been recognized early on that the accuracy of the measurements of small anomalous differences, and therefore the chances of solving the corresponding structure by S-SAD phasing, can be improved by collecting data with high multiplicity (Dauter & Adamiak, 2001 ▸; Weiss *et al.*, 2001 ▸). However, even with the most accurate experimental apparatus, the collection of high-multiplicity diffraction data from a macromolecular crystal is limited by X-ray radiation damage (Garman & Weik, 2017 ▸).

X-ray radiation damage to macromolecular crystals is observable in reciprocal space by the fading of Bragg reflections starting with those at high resolution (Blake & Phillips, 1962 ▸; Hendrickson, 1976 ▸; Holton, 2009 ▸). Inside the crystal, radiation damage typically manifests itself, first in the reduction of metal centers, followed by cleavage of di­sulfide bonds and the de­carboxylation of aspartates and glutamates (Garman & Weik, 2015 ▸). The increasing effects of radiation damage on a macromolecular crystal can be related to the absorbed X-ray energy per unit mass, *i.e.* the dose, and the computer program *RADDOSE-3D* (Zeldin *et al.*, 2013 ▸) has been developed to estimate the dose deposited in a crystal as a function of the X-rays used and the composition of the sample. With *RADDOSE-3D*, the approximate life-time of a macromolecular crystal in a diffraction experiment can be predicted and used to optimize the collection of native data. For the quantification of the actual effects of radiation damage on the diffraction signal from a crystal, a number of metrics are available [see Garman (2010 ▸) for a review], while the detailed consequences of radiation damage can be observed in crystal structures (Burmeister, 2000 ▸; Leiros *et al.*, 2001 ▸; Schiltz *et al.*, 2004 ▸). The effects of specific radiation damage on the structure factor amplitudes measured from a crystal have been discussed by Owen & Sherrell (2016 ▸).

In the context of collecting anomalous diffraction data for the purpose of SAD phasing, the relation between radiation damage as macroscopically observable by changes in diffracted intensities and the usability of the anomalous signal is not obvious. Although the chemical reactions involving anomalous scatterers such as the breakage of disulfide bridges are in principle known, the dependence of their progression on the absorbed X-ray dose is strongly dependent on the chemical environment of the respective atoms, rendering predictions difficult.

For S-SAD structure determinations where relatively large crystals are available, low-dose high-multiplicity data collection techniques have been advocated (Weinert *et al.*, 2015 ▸; Olieric *et al.*, 2016 ▸). These techniques exploit the fact that for modern photon-counting and virtually noise-free X-ray detectors the total tolerable dose can be spread over many frames and the signal-to-noise for the measured intensities can be improved by assembling measured intensities by summing (symmetry-) equivalent reflections. Data can then be truncated, if necessary by trial-and-error, *post factum* as a function of applied dose in order to optimize data quality. Such truncation is in fact particularly important in the case of preparing data for SAD phasing, as the inclusion of inaccurate data can, despite the gain in data precision due to increased multiplicity, do more harm than good in the subsequent substructure determination and phasing steps. In practice, doses of the order of 4–5 MGy have been found to limit the damaging effects of radiation to the tolerable with respect to S-SAD phasing (Weinert *et al.*, 2015 ▸; Olieric *et al.*, 2016 ▸; Liu *et al.*, 2014 ▸).

Here, we address the problem of identifying the point of diminishing returns in high-multiplicity S-SAD phasing, *i.e.* the point at which the inclusion of additional data from a progressively more damaged crystal leads to a deterioration in the quality of the substructure determined from these data, from only experimental diffraction data in a systematic fashion by correlating statistical properties of the measured anomalous differences with the quality of the substructure obtained.

## 2. Materials and methods   

### 2.1. Crystals   

Thaumatin (UniProt: P02883) is a sweet-tasting protein from *Thaumatococcus daniellii* containing 207 amino acid residues including a total of 17 S atoms (1 in a me­thio­nine and 16 in cysteine residues). In its folded form, the protein forms eight di­sulfide bonds. At an X-ray energy of 8 keV, *f*′ is 0.56 electrons (http://skuld.bmsc.washington.edu/scatter/AS_index.html) resulting in an expected Bijvoet-ratio of approximately 1.2% (Smith, 1991 ▸). Thaumatin was purchased as a powder from Sigma Aldrich and dissolved in 0.1 *M* BIS-TRIS propane at pH 6.5 to a concentration of 48 mg ml^−1^. Crystals were grown by the hanging drop method in 24-well Limbro-plates on glass cover slides by mixing 1 µl of protein solution with 1 µl of well solution containing 0.1 *M* BIS-TRIS propane and 0.6–1 *M* sodium tartrate. At room temperature, crystals appeared within two days.

### 2.2. Beam conditions   

**Figure 1:**
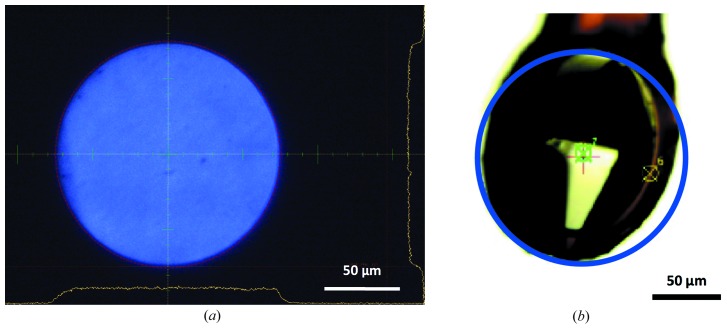
(*a*) Profile of the unfocused X-ray beam on beamline P14 after passing through a 150 µm-diameter circular aperture as imaged on the scintillator crystal of the MD3. (*b*) Crystal *A* mounted in a lithographic loop. The blue circle indicates the dimensions of the X-ray beam.

All diffraction data were collected on EMBL beamline P14 on the PETRA III storage ring at DESY (Hamburg, Germany) at an X-ray energy of 8.01 keV employing ‘unfocused’ or ‘collimated’ conditions. For unfocused conditions, no focusing optics are used in the beamline while, for collimated conditions, compound refractive lenses (Snigirev *et al.*, 1996 ▸) mounted in a transfocator (Snigirev *et al.*, 2009 ▸; built by CINEL, Vigonza, Italy) in the white beam upstream of the monochromator are used to collimate the X-ray beam. For both conditions the beam profile at the sample position is highly homogeneous (Fig. 1 ▸).

For both conditions, the total photon flux through a 150 µm-diameter circular aperture as available on the MD3 diffractometer (ARINAX, Moirans, France) was measured using a calibrated pin diode. Measurements taken immediately before and immediately after groups of diffraction data collections resulted in total photon fluxes of 5.5 × 10^10^ photons s^−1^ and 5.2 × 10^10^ photons s^−1^ (corresponding to a ∼6% decrease during 11 h) for the unfocused and 1.05 × 10^12^ photons s^−1^ and 4.98 × 10^11^ photons s^−1^ (corresponding to a 53% decrease during 8.5 h) for the collimated beam, respectively. Taking into account that collection of a full 360° wedge took ∼6 min under unfocused and ∼36 s under collimated conditions (see below), the observed intensity variations do not play a significant role in our analysis.

### 2.3. Data collection   

For data collection, crystals were cryo-protected by adding a solution containing 0.6 *M* sodium tartrate, 0.1 *M* BIS-TRIS propane and 25% glycerol to the crystallization drop, mounted in a lithographic loop (Mitegen, Ithaca, NY, USA) and cooled in a stream of gaseous nitrogen at 100 K. Crystals were selected and mounted such that they could be fully bathed in the circular X-ray beam of 150 µm diameter (Fig. 1 ▸). Diffraction data were collected from three crystals (*A*, *B*, *C*) under unfocused conditions on 21/4/2015 and from two crystals (*D*, *E*) under collimated conditions on 27/9/2015 at an X-ray energy of 8.01 keV. Crystals were rotated continuously using the vertical-spindle MD3 diffractometer on P14 and rotation exposures with an oscillation range of 1° were recorded in shutterless mode on a PILATUS 6M detector (Dectris AG, Baden, Switzerland). Exposure times for crystals *A*, *B*, *C* were 1 s deg^−1^ (*i.e.* 6 min data collection time per 360° wedge) while, for crystals *D* and *E*, 0.1 s deg^−1^ (*i.e.* 36 s per 360° wedge) were used. At a crystal-to-detector distance of 152.4 mm, data to a maximum resolution of 1.7 Å were recorded for between 10 and 20 full 360° rotations of the crystal.

### 2.4. Dose estimation   

Assuming a homogeneous beam profile, and flux values determined by linear interpolation between values measured at the beginning and at the end of a group of data collections (see above), average diffraction weighted doses (referred to as ‘dose’ in the following) were estimated for each dataset with *RADDOSE-3D* (Zeldin *et al.*, 2013 ▸); for values see Table 1 ▸.

### 2.5. Data processing and (sub)structure determination   

Each 360° wedge of diffraction data was integrated separately with *XDS* (Kabsch, 2010 ▸) using standard parameter settings and written to file without merging symmetry-equivalent reflections. The space group for all datasets was *P*4_1_2_1_2. For each crystal, measured reflection intensities were, after modifying image numbers using a custom Python-script, accumulated into files containing data collected with increasing numbers of consecutive 360° wedges, *i.e.* files containing data obtained from 0–360°, 0–720°, 0–1080°,… rotations of the respective crystals. We denote these datasets as ‘accumulated’ datasets. It should be noted that for the accumulated datasets no relative scaling between the wedges was applied at this stage.

### 2.6. Structure solution and refinement   

Data from individual wedges and the accumulated data were further analyzed and processed with *SHELXC* (Sheldrick, 2010 ▸). Sulfur substructures were determined with *SHELXD* (Schneider & Sheldrick, 2002 ▸) searching for nine sites, including eight super atoms, which would then be split into individual S atoms against anomalous differences to a maximum resolution of 2.8 Å (SHEL 999 2.8, FIND 9, DSUL 8, NTRY 10000). During substructure solution it became apparent that the best solutions found by *SHELXD* against accumulated wedges systematically exhibited higher CFOM values when no relative scaling between the wedges was applied (data not shown). All subsequent analysis was therefore performed on data to which no relative scaling between wedges was applied at the *XDS* stage. However, it should be noted that *SHELXC* does apply a local scaling procedure for the determination of signed anomalous differences from incoming unmerged data.

*SHELXE* (Sheldrick, 2010 ▸) was used for substructure refinement and initial phasing, followed by three rounds of 20 cycles of density modification with a solvent fraction of 0.539 in combination with automatic main chain-tracing (-m20 -s0.539 -h -z -a3). The model obtained was completed in ten building cycles in *Arp/Warp* (Lamzin *et al.*, 2012 ▸; Winn *et al.*, 2011 ▸). For each crystal, the first accumulated dataset for which a CFOM comparable with the absolutely highest CFOM was initially reached in the substructure determination by *SHELXD* was selected as the target for a refinement of a reference model (Table 1 ▸). This choice was made since for some crystals the highest CFOM was reached at a dose at which the determined substructure would no longer correspond to the substructure at the beginning of the experiment due to radiation damage effects. Refinement, manual model building and Ramachandran statistics were carried out with *Refmac5* (Murshudov *et al.*, 2011 ▸) and *Coot* (Emsley *et al.*, 2010 ▸).

### 2.7. Substructure validation   

To access the correctness of sulfur substructures obtained by *SHELXD* against different diffraction datasets, the substructures were compared with the S atoms in the respective refined reference structure using *SITCOM* (Dall’Antonia & Schneider, 2006 ▸).

### 2.8. Calculation of anomalous differences   

Signed anomalous difference estimates 〈Δ*F*〉 were determined using a custom space-group specific Python-program as
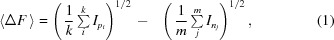
based on the reflection intensities of all Bijvoet-positives *I*_P*i*_ and all Bijvoet-negatives *I*_Ni_ related to a unique Friedel-pair. Intensities measured as negative were ignored. In the following, 〈Δ*F*〉 values as determined by equation (1) are denoted as *Δ* *F* values.

### 2.9. Calculation of the σ{Δ*F*} metric   

For the characterization of the distributions of Δ*F* values determined following equation (1), histograms were fitted with Gaussian distributions 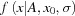 = 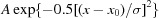 employing 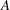, 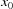 and 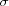 as fitting parameters. For determining the σ{Δ*F*} metric for a specific subset of data, the Δ*F* histograms were converted into frequency distributions by dividing all bins by the total number of anomalous differences observed for the respective wedge. The frequency distributions were then fitted by normal distributions, 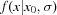 = 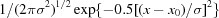, employing 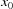 and 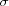 as fitting parameters. Here it should be noted that the variation in the number of Δ*F* values obtained from different wedges or accumulated subsets of data for a given crystal was less than 3–4% for all cases, and considered to be negligible in the determination of σ{Δ*F*} values. All fits were performed using functions available in the Python NumPy library.

## 3. Results and discussion   

### 3.1. Data and structure quality   

The crystals used for data collection showed comparable mosaicities between 0.07 and 0.19° (in *XDS* units) for the first 360° wedge of data collected. The data collected are more than 98% complete in all cases and of excellent quality exhibiting high signal-to-noise ratios and low merging *R* values (Table 1 ▸).

**Figure 2:**
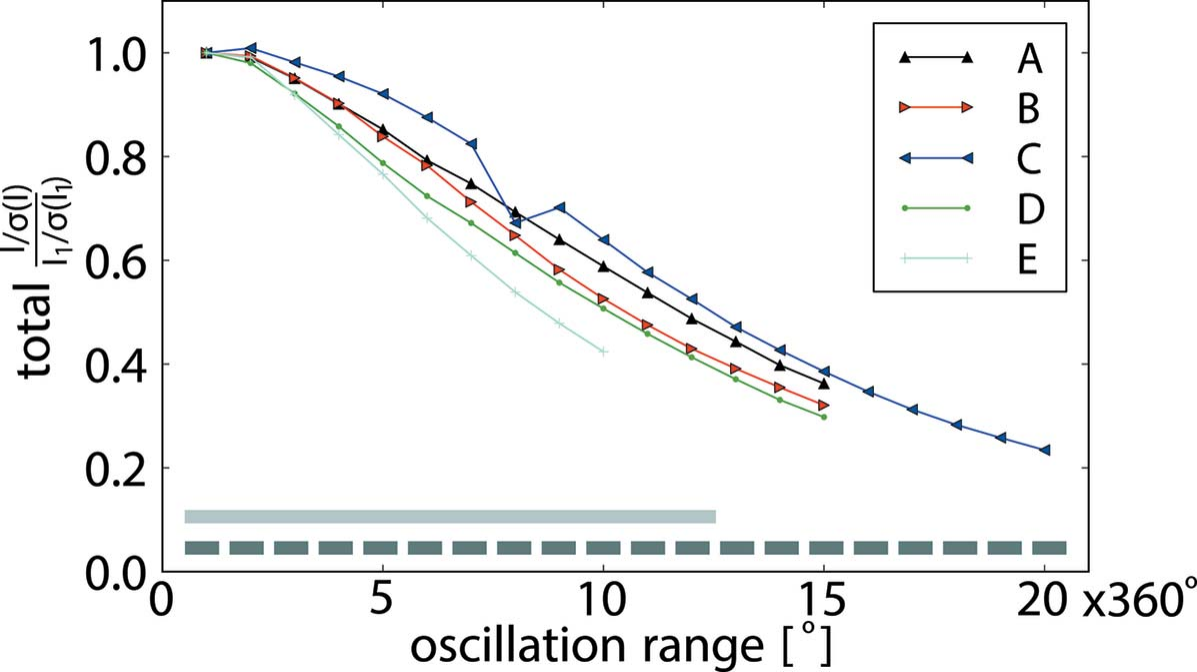
〈*I*/σ(*I*)〉, normalized to 〈*I*_1_/σ(*I*_1_)〉 as observed for the first wedge, as a function of oscillation range for crystals *A* (black), *B* (red), *C* (blue), *D* (green), *E* (turquoise). Values determined for 〈*I*_1_/σ(*I*_1_)〉 for the different crystals are given in Table 1 ▸. Each data point corresponds to the respective separately processed 360° wedge. ‘Accumulated data’ employed for parts of the analyses described in the following correspond to groups of consecutive wedges merged together (indicated by the light grey bar at the bottom of the plot corresponding to data merged from the first 12 turns of data collection).

The decay of the normalized average signal-to-noise for the diffracted intensities 〈*I*/σ(*I*)〉 as a function of dose (Fig. 2 ▸) is similar for all five crystals and shows, as expected for diffraction data recorded at cryogenic temperatures, an exponential decay, indicating progressing radiation damage (Bourenkov & Popov, 2010 ▸). The point at which 〈*I*/σ(*I*)〉 drops to approximately half of its initial value is reached after 8–12 full rotations of the crystals in the X-ray beam corresponding to an approximate dose of 5–8 MGy absorbed by the crystals. The differences in slope of the decay curves can be attributed firstly to the different high-resolution cut-offs employed for the different datasets: as high-resolution reflections will fade faster than low-resolution reflections with progressing radiation damage, 〈*I*/σ(*I*)〉 for the high-resolution datasets (*D*, *E*) will be affected earlier than for the lower-resolution datasets (*A*, *B*). In addition, it cannot be excluded that the crystals were irradiated at different X-ray dose rates, since the X-ray flux of the beamline, for technical reasons, was calibrated only some hours before and after the measurements shown were taken. For crystal *C*, 〈*I*/σ(*I*)〉 at the beginning of the data collection (Table 1 ▸) is significantly higher than for crystals *A*, *B*, *D* and *E*, reflecting the fact that crystal *C* has a significantly larger volume than the other crystals. The refined reference structures are of high quality as indicated by the *R*_work_ and *R*_free_ statistics, the stereochemical parameters and the agreement with the expected distributions of Ramachandran angles (Table 1 ▸).

### 3.2. Identification of the most accurate substructure   

**Figure 3:**
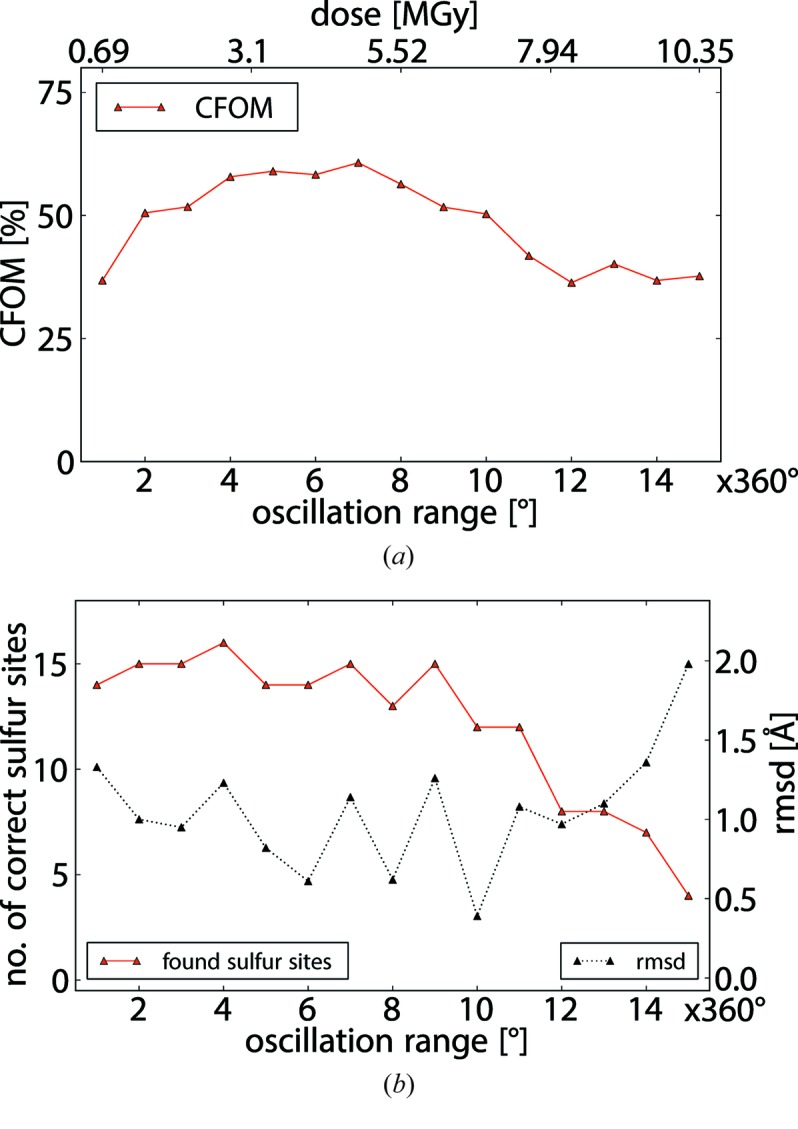
Substructure quality for dataset *A*. (*a*) CFOM for the best substructure solution obtained by *SHELXD* as a function of total oscillation range. (*b*) The number of correct sulfur sites (red) and the corresponding r.m.s.d. (dashed grey) with respect to the reference structure *A* as determined by *SITCOM*. Here, the most accurate substructure is identified as the one for which 4 × 360° of accumulated data are used.

Sulfur substructures were determined against different accumulated subsets of the data collected on each crystal. For each subset of data, CFOM values as obtained by *SHELXD* and the number of sites consistent with the sulfur sites in the reference structures were analyzed (Fig. 3 ▸). For all datasets, the substructure could be solved, with CFOM values above 50 and a maximum of 16 correctly identified sulfur sites.

For the initial phase of all data collections, the increased multiplicity of the raw data results in more accurate estimates for the anomalous differences leading to higher-quality substructure solutions as reflected in higher CFOM values (Fig. 3 ▸). However, in later phases of the data collections the anomalous differences obtained become less accurate due to progressing radiation damage, as indicated by a decrease in the CFOM values as a function of total dose absorbed by the crystal.

For each crystal, subset(s) of data for which the highest-quality substructure solution was determined were identified by taking into account the CFOM values, the number of correctly identified sites and the r.m.s.d. with respect to the reference structure. For four (*A*, *B*, *D*, *E*) out of the five crystals analyzed, the number of wedges collected to achieve an optimum substructure solution is three to four, while for crystal *C* the number of respective wedges is about twice as large (Table 2 ▸). In fact, crystal *C* has about four times the volume exposed to beam in comparison with the other crystals. As a larger volume, in comparison with a smaller volume, inherently leads to a higher signal-to-noise at the beginning of and during the data collection, the deterioration of the quality of the measured diffraction intensities will affect the quality of the derived anomalous differences in a way that outbalances the gains from high multiplicity only at higher doses than for smaller crystals.

### 3.3. Anomalous differences   

**Figure 4:**
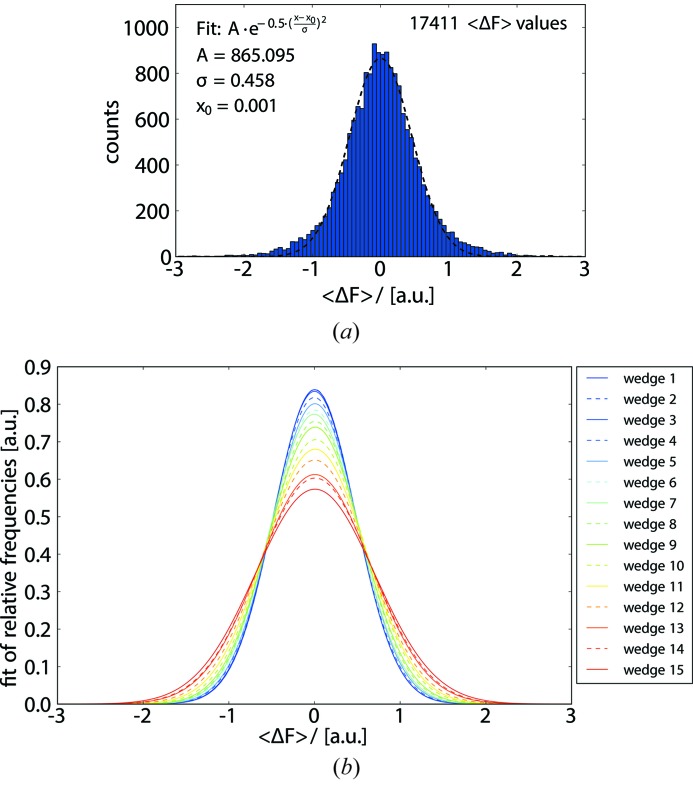
Distributions of Δ*F* values. (*a*) Histogram of Δ*F* values obtained from the first 360° wedge collected on crystal *A* (blue bars), {Δ*F*}_1_, fitted with a Gaussian (dashed line); fitting parameters are shown in the inset. Note that the fraction of Δ*F* values outside the interval [−3, +3] (not shown in the graph) is less than 0.1% of the total number of the Δ*F* values. (*b*) Gaussians obtained by fitting the frequency distributions obtained for {Δ*F*}_1_ to {Δ*F*}_15_, respectively, using colour codes defined in the inset. The functions used for fitting are defined in §2.9.

To establish a metric capable of guiding the selection of a sub-dataset producing the best substructure solution without actually knowing the substructure beforehand, we analyzed various statistics on averaged signed anomalous differences for differently chosen subsets of reflections {Δ*F*}. For histograms of Δ*F* we found that these are generally bell-shaped, consistent with the prediction of Ursby & Bourgeois (1997 ▸) that the anomalous differences should have a Gaussian distribution. Visual inspection of the histograms as a function of dose revealed that, at the beginning of a data collection, the distributions are relatively sharp and become broader with increasing dose (Fig. 4 ▸).

**Figure 5:**
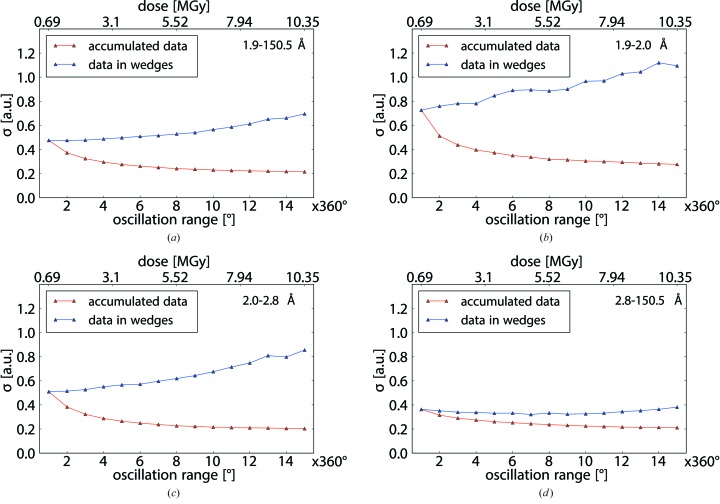
σ{Δ*F*}_*i*_ (blue triangles) and σ{Δ*F*}_acc,*i*_ (red triangles) as a function of wedge number *i* and dose (top) for different resolution ranges for crystal *A*. (*a*) All data. (*b*) High-resolution data from 1.9 to 2.0 Å. (*c*) Medium-resolution data from 2.0 to 2.8 Å. (*d*) Low-resolution data from 2.8 to 150.5 Å.

**Figure 6:**
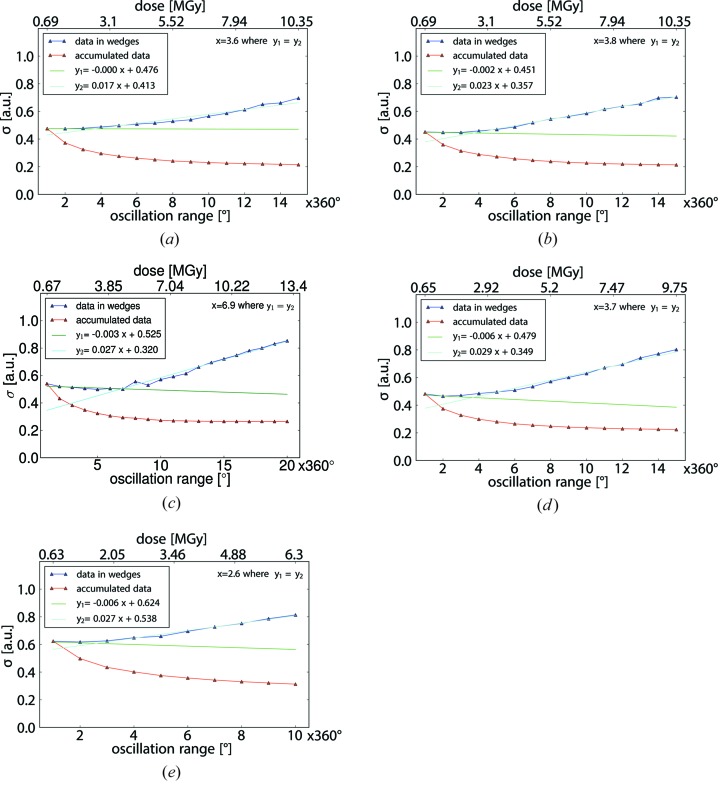
Plots of σ{Δ*F*} against the number of 360° turns (bottom) and estimated X-ray dose (top) for all reflections for crystals *A*, *B*, *C*, *D*, *E* [panels (*a*), (*b*), (*c*), (*d*), (*e*)]. Fitting parameters are indicated in the insert in the top left; the crossing point of the two fits is shown in the top right corner of each panel.

For quantitative comparative analysis, distributions of Δ*F* originating from individual wedges *i *{Δ*F*}_*i*_ or from data accumulated up to a wedge number *i* {Δ*F*}_acc,*i*_ were normalized and fitted with normal distributions to determine their means and widths σ{Δ*F*}. The widths σ{Δ*F*} were plotted as a function of dose (Figs. 5 ▸ and 6 ▸). For all five crystals, similar behaviours of σ{Δ*F*} as a function of dose were observed. While for accumulated data, σ{Δ*F*}_acc,*i*_ decreases strictly monotonically, the σ{Δ*F*}_*i*_ values determined for individual wedges initially remain constant or decrease and at a certain point begin to increase strictly monotonically (Fig. 6 ▸).

This behaviour can be explained by assuming that the observed distributions are convolutions of the distribution of the true anomalous differences and the distribution of the associated errors. Assuming that, to a first approximation, the relative error in the measurement of intensities is mostly linked to counting statistics, the relative error of the measured intensities σ_*I*_/*I* will increase as a function of dose as intensities will decrease due to the progressing loss of crystalline order caused by radiation damage. Consequently, the relative error on Δ*F* will increase and be reflected increasingly more strongly in a broadening of the distributions of Δ*F* values.

The overall variance of an intensity measurement σ^2^ can be approximated as σ^2^ = 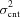 + *KI* ^2^ (Leslie, 1999 ▸), where σ_cnt_ represents the variance from Poisson counting statistics including background noise and *KI* ^2^ corresponds to errors proportional to the intensity such as time-dependent variations in X-ray intensity, spatial variations in detector efficiency and other factors. The relative contribution of the 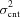 and the *KI* ^2^ terms to the overall variance σ^2^ will shift towards 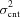 while the diffraction intensities are decreasing. The dose at which 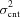 will become the dominating contribution will depend on the initial intensity level and the quality of the experimental apparatus reflected in the constant *K*. For ‘overdosed’ experiments, the *KI* ^2^ term may be the dominating one during the initial phase of the experiment. The small negative slope of σ{Δ*F*}_*i*_ at the beginning of some data collections is mostly due to a decrease of the scattering power of the substructure as a consequence of specific radiation damage.

For accumulated data, the relative errors in the measurement of Δ*F* are in addition reduced by a factor of *N* ^−1/2^ when *N* multiple measurements on (symmetry-) equivalent reflections are taken. Therefore, both the Poissonian and the instrument contribution to the relative measurement errors will decrease during the initial phase of the data collection, resulting in a sharpened distribution for the measured Δ*F* values. The observed improvement in the substructure quality (Fig. 3 ▸) during the early phase of data collection indicates that, overall, the loss of anomalous scattering signal is smaller than the gain in data quality due to increased multiplicity.

Based on the above observations, we propose that the effect of radiation damage on the quality of anomalous difference can be measured by evaluating σ{Δ*F*} for independent groups of reflections as the deposited X-ray dose increases. Thus, σ{Δ*F*}_*i*_ can be considered as a potential metric for the influence of radiation damage on the quality of measured anomalous differences.

### 3.4. Resolution dependence   

To further investigate the behaviour of σ{Δ*F*}_*i*_ as a function of dose, we inspected data from different resolution shells separately (Fig. 5 ▸). For low-resolution reflections, σ{Δ*F*}_*i*_ starts off at 0.38, which is substantially smaller than the corresponding value of 0.58 for all data, indicating that the width of the Δ*F* distribution for strong reflections is less affected by measurement errors than the distribution for all reflections. Indeed, in the dose regime considered here, low-resolution reflections should not be significantly affected by radiation damage (Howells *et al.*, 2009 ▸; Bourenkov & Popov, 2010 ▸).

In contrast, for high-resolution reflections, which are expected to be affected more rapidly by radiation damage (Blake & Phillips, 1962 ▸; Hendrickson, 1976 ▸; Leal *et al.*, 2011 ▸), the initial value of σ{Δ*F*}_*i*_ is higher than for all data, and the increase in σ{Δ*F*}_*i*_ is observed at lower dose. This reflects the diminution of the overall diffraction signal due to global damage and possibly also due to variations in the structure factors caused by actual structural changes induced by X-ray irradiation.

For intermediate resolution, a range of 2.0 to 2.8 Å was chosen because experience has shown that data quality in this shell of reciprocal space is often decisive for the success of S-SAD phasing. The behaviour of reflections from the intermediate resolution shell corresponds to the behaviour for all data.

These observations for groups of weak, intermediate and strong reflections support the above argument about the varying contributions of instrumental and counting errors to measured anomalous differences as a function of X-ray dose.

### 3.5. Determination of the limiting σ{Δ*F*}_*i*_ as a dose-dependent metric for anomalous data quality   

While the plots of σ{Δ*F*}_acc,*i*_ for the accumulated data subsets are strictly monotonically decreasing, the plots of σ{Δ*F*}_*i*_ show a characteristic change in slope as a function of dose. The transition point between the different slopes can be detected by fitting straight lines to the respective monotonically decreasing and the monotonically increasing subsets of data points (Fig. 6 ▸). Comparison of the identified transition points with the sub-datasets resulting in an optimum substructure reveals a strong correlation between the two (Table 3 ▸). This indicates that the dose at which the slope of σ{Δ*F*}_*i*_ *versus* dose changes could be used as a predictor for the dose at which the highest-quality anomalous differences can be extracted for a given crystal for which diffraction is observed on a given instrument.

The transition points for crystals *A*, *B*, *D* and *E* are similar in terms of an absorbed dose of 2–3 MGy, while for crystal *C* the transition takes place at a significantly higher dose of 5 MGy. This observation can be attributed to the facts that (i) the overall 〈*I*/σ(*I*)〉 at the beginning of a data collection is naturally higher for a large crystal than for a small crystal (see Table 1 ▸) and (ii) the initial slope of 〈*I*/σ(*I*)〉 is less for a larger crystal as the instrument contribution to the error, which is independent of the absorbed dose, is dominating. Thus, despite the fact that the same dose damages the crystal in the same way (in terms of intensity loss and specific damage), regardless of its size, a higher dose can be absorbed by a larger crystal before *I*/σ(*I*) becomes intolerably low.

## 4. Conclusions and perspectives   

Based on the analysis of SAD phasing from five high-multiplicity datasets as collected from five different thaumatin crystals, we propose the standard deviation of the normalized distribution of anomalous differences as a function of estimated X-ray dose absorbed by a crystal, termed σ{Δ*F*}_*D*_, as a metric for assessing the effects of radiation damage on the anomalous data collected for sulfur SAD phasing. Using the correctness of substructures obtained as a function of dose as a guide, a procedure is suggested to identify the dose at which the gains in data quality from increased multiplicity are balanced by the losses in data quality due to radiation damage.

σ{Δ*F*}_*D*_ is a purely experimental measure that can be determined rapidly during an on-going measurement. Its calculation does not require any assumptions about the processes taking place inside the crystal when it is exposed to X-rays. For the case of S-SAD phasing of thaumatin crystals, we found that the point of diminishing returns in terms of anomalous signal quality can be identified by an analysis of the course of σ{Δ*F*}_*D*_ and is reached for an absorbed diffraction weighted dose between 1.6 and 4.6 MGy. For larger crystals, larger doses seem to be tolerable due to the inherently higher diffraction signal-to-noise ratio for a large crystal in comparison with a small crystal. The observations on the large crystal in this study are in quantitative agreement with previous studies, where for large (linear dimensions in the 50–300 µm range) crystals a dose limit for anomalous data collection of approximately 5 MGy was found (Weinert *et al.*, 2015 ▸; Olieric *et al.*, 2016 ▸; Liu *et al.*, 2014 ▸).

An analysis of σ{Δ*F*}_*D*_ during an on-going data collection can be used to guide the choice of an optimum subset of data collected to drive rapid structure solution procedures as the ultimate validation of the data being collected. More sophisticated procedures than the simplistic one presented here could be applied.

Preliminary experiments concerning S-SAD phasing on Zn-free insulin and lysozyme crystals (data not shown), both proteins containing di­sulfides, have shown a qualitatively similar behaviour to the observations on thaumatin crystals presented here. It will also be of interest to analyze crystals containing anomalous scatterers other than sulfur to find out whether a similar approach would be applicable. For example, in trypsin, the structural effects of radiation damage (de­carboxyl­ation of a glutamate side-chain coordinating a Ca^2+^ ion; Schroeder-Leiros *et al.*, 2001 ▸) are markedly different from the breaking of di­sulfide bonds. If signals from low-resolution reflections are used for phasing, *e.g.* in cluster-based phasing, the tolerable X-ray doses may be much larger than for high-resolution phasing, given the higher robustness of low-resolution reflections against radiation damage. Nevertheless, the σ{Δ*F*}_*D*_ metric could be applicable in these cases as, for its determination, no assumptions are made about the specific nature of the processes taking place.

For calculation of the σ{Δ*F*}_*D*_ metric, low-dose high-multiplicity data collections are of advantage as these will provide statistically more robust and more fine-grained estimates of σ{Δ*F*} as a function of dose than low-multiplicity datasets. Provided that stable X-ray beams, fast and reliable diffractometers and low-noise detectors are available, such data collections optimizing the chances of success for S-SAD structure solution can be performed robustly on time-scales comparable with ‘faster’ experiments which use higher dose rates.

## Figures and Tables

**Table 1:** Data collection and structure phasing All data were collected at an X-ray energy of 8.01 keV. Values for the highest-resolution shells are given in parentheses. ‘Wedge 1’ relates to data collected between 0 and 360° rotation of the crystal. All data statistics refer to datasets in which Friedel-related reflections were treated separately. 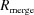 = 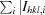 − 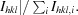 ‘CFOM_phs_’ relates to the sub-dataset indicated in the ‘Data’ row which was used for structure solution and refinement. *N*_res,shelxe_ and *N*_res,arp_ refer to the number of residues built automatically by *SHELXE* and *Arp/Warp*, respectively. CC_partial_ is the correlation coefficient for the partial structure built by *SHELXE* against the native data.

Crystal	*A*	*B*	*C*	*D*	*E*
Data collection					
Flux (photons s^−1^)	5.5 × 10^10^	5.5 × 10^10^	5.5 × 10^10^	5.1 × 10^11^	5.1 × 10^11^
Exposure (s deg^−1^)	1.0	1.0	1.0	0.1	0.1
Crystal size (µm)	100 × 50 × 55	110 × 60 × 40	145 × 90 × 90	130 × 64 × 52	152 × 78 × 60
Unit cell (*a* = *b*) (Å)	57.82	57.71	57.83	57.86	57.74
*d*_min_ (Å)	1.9 (2.02–1.90)	1.9 (2.02–1.90)	1.75 (1.85–1.75)	1.7 (1.8–1.7)	1.7 (1.80–1.70)
No. of 360° turns	15	15	20	15	10
Dose/360° (MGy)	0.69	0.69	0.67	0.65	0.63
					
Data processing Wedge 1					
No. of collected reflections	448093	498286	575804	624231	620165
No. of unique reflections	38382	37704	48432	53610	52946
Mosaicity (°)	0.12	0.16	0.16	0.07	0.19
Completeness (%)	99.9 (99.5)	98.6 (97.1)	98.0 (88.3)	99.9 (99.5)	98.9 (95.2)
*R*_merge_ (%)	7.6 (41.2)	7.6 (35.3)	5.4 (29.6)	7.2 (30.9)	6.7 (47.8)
〈*I*/σ_*I*_〉	28.9 (6.5)	31.5 (8.0)	35.4 (6.7)	24.8 (6.5)	29.1 (4.8)
					
Structure solution					
CFOM_phs_ (%)	63.3	68.9	74.8	54.6	63.0
Data (°)	2 × 360	3 × 360	5 × 360	2 × 360	1 × 360
Multiplicity	25.5 (18.6)	38.4 (28.9)	56.5 (20.4)	22.6 (14.3)	11.6 (7.9)
*N*_res,shelxe_	203	191	195	192	182
CC_partial_ (%)	48.2	43.9	45.3	42.7	40.3
*N*_res,arp_	203	205	204	201	205

**Table 2:** Refinement For refinement, the sub-datasets used for phasing were selected and scaled together with *AIMLESS* (Evans & Murshudov, 2013 ▸; Winn *et al.*, 2011 ▸). All statistics refer to Friedel-pairs merged. Values for the highest-resolution shells are given in parentheses. For Ramachandran statistics, the percentages of preferred, acceptable and outlier residues are given.

Crystal	*A*	*B*	*C*	*D*	*E*
Data statistics					
Resolution	1.9 (1.94–1.90)	1.9 (1.94–1.90)	1.75 (1.78–1.75)	1.70 (1.73–1.70)	1.70 (1.73–1.70)
No. of collected reflections	1010376	1498152	2889189	1250117	620176
No. of unique reflections	21064	20641	26561	29140	28645
Completeness (%)	99.9 (98.3)	99.2 (97.3)	98.4 (72.1)	99.9 (98.6)	99.1 (93.8)
*R*_merge_ (%)	8.6 (55.8)	9.6 (50.7)	8.4 (46.4)	8.2 (40.3)	6.9 (59.0)

Refinement					
*R*_work_ (%)	16.2	16.5	15.2	16.1	15.7
*R*_free_ (%)	18.1	19.5	18.0	18.5	18.6
r.m.s.d. bond lengths (Å)	0.053	0.034	0.031	0.042	0.035
r.m.s.d. bond angles (°)	3.40	2.34	2.78	2.59	2.32
Ramachandran (%)	97.1, 2.4, 0.5	98.5, 1.5, 0.0	98.0, 2.0, 0.0	97.5, 2.0, 0.5	97.5, 2.0, 0.5

**Table 3:** σ{Δ*F*}_*D*_ and highest quality substructures Data_best_ denotes the range of data from which the most accurate substructure was determined. Data_σ*_ denotes the range of data for which the transition point between the early and the asymptotic slope of σ{Δ*F*}_*i*_ as a function of dose was determined.

Crystal	*A*	*B*	*C*	*D*	*E*
Data_best_ (°)	4 × 360	2–7 × 360	7 × 360	4 × 360	3 × 360
Data_σ*_	3.6 × 360	3.8 × 360	6.9 × 360	3.7 × 360	2.6 × 360
